# Astrocyte elevated gene 1: biological functions and molecular mechanism in cancer and beyond

**DOI:** 10.1186/2045-3701-1-36

**Published:** 2011-11-07

**Authors:** Zhe Ying, Jun Li, Mengfeng Li

**Affiliations:** 1 Department of Microbiology, Zhongshan School of Medicine, Sun Yat-sen University, Guangzhou, Guangdong 510080, China; 2 Department of Biochemistry, Zhongshan School of Medicine, Sun Yat-sen University, Guangzhou, Guangdong 510080, China; 3 Key Laboratory of Tropical Disease Control (Sun Yat-sen University), Chinese Ministry of Education, Guangzhou 510080, China

## Abstract

Since its discovery, nearly one decade of research on astrocyte elevated gene 1 (AEG-1) has witnessed expanding knowledge of this molecule, ranging from its role in cancer biology to molecular mechanisms underlying the biological functions. As a multifunctional oncoprotein, AEG-1 has been shown to overexpress in multiple types of human cancer, and the elevation of AEG-1 in tumor cells leads to enhanced phenotypes characteristic of malignant aggressiveness, including increased abilities to proliferate robustly, to invade surrounding tissues, to migrate, to induce neovascularization, and to enhance chemoresistance. The multifunctional role of AEG-1 in tumor development and progression has been found to be associated with several signaling cascades, namely, 1) activation of NF-kappa B, partially through direct interaction with p65; 2) PI3K/AKT signaling triggered by AEG-1 indirectly; 3) enhancement of the transcriptional activity of beta-catenin by indirect activation of MAPK and induction of LEF1; 4) regulation of mi/siRNA-mediated gene silencing by interacting with SND1; and 5) promotion of protective autophagy; in addition to possibly unknown mechanisms. Elevated AEG-1 expression is seen in nearly all tumor types, and in most cases AEG-1 positively correlates with tumor progression and poorer patient survival. Taken together, AEG-1 might represent a potential prognostic biomarker and therapeutic target.

## Discovery and molecule characteristics of AEG-1

In 2002 and 2004, several groups, under different experimental settings, reported identification of the gene, first named as astrocyte elevated gene 1 (AEG-1), now having the GenBank symbol as MTDH, which stands for metadherin, for its involvement in tumor metastasis and adhesion. In an attempt to identifying molecules involved in mediating HIV-related neuron degeneration, Fisher's group, by using subtraction hybridization, found AEG-1 as a novel transcript induced by HIV-1 infection or gp120 treatment in human fetal astrocytes [1]. In another study by Brown and Ruoslahti in 2004, phage display strategy was employed in a mouse model of breast cancer metastasis, which revealed that a gene identical to AEG-1 was expressed at high levels in relation to metastasis of breast cancer, and the identified gene was named metadherin after its proposed role in promoting homing of breast cancer cells to the lungs [2]. In the same year, Britt et al. and Heidi et al., independently, identified that the same molecule, this time designated LYRIC in both cases, was a novel component of the tight junction structure of epithelial cells [3] and an endoplasmic reticulum/nuclear envelope-associated protein [4], respectively.

While the initial discovery of AEG-1 left with an abundance of controversial understanding of its biological functions and biochemical characteristics, which remains elusive to date, a few features of AEG-1 have been recognized with certain consensus. In human, AEG-1 represents a protein of 582 amino acids, and the amino acid sequences are highly conserved across vertebrates. Despite its conservativeness, however, the three-dimensional structure of AEG-1 has not been solved, and the functional domains of the protein are not clearly defined. To understand the biological functions of AEG-1 in cells, however, its localization has been investigated. In previously published studies, the intracellular localization of AEG-1 appeared to be variable and depend on the cell types examined and the imaging techniques employed. In most cases both endogenous or ectopically expressed AEG-1 is detectable in cytoplasm as well as in the nucleus by immunohistochemical and immunofluorescent staining of cultured cells or sectioned specimens of tissues [4-7]. In rat liver, however, AEG-1 was displayed on cell membrane [3], whereas GFP-fused AEG-1 showed stronger staining in the nucleolus [8,9]. Three putative NLS has been predicted in the lysine-rich regions of AEG-1 [4,6]. In the latter report, interestingly, the functional importance of these three regions was examined, and by using the GFP reporting system, NLS 1 or 3 and their flanking regions was shown to be able to target the reporter GFP peptide to nuclear and nucleolus [9]. Moreover, as a protein rich of the lysine residues, AEG-1 could be potential subject to post-translational ubiquitination, SUMOlation or acetylation [6]. There has been evidence indicating potential mono-ubiquitination of AEG-1 in its NLS2 sequence and the flanking regions, and ubiqutinated AEG-1 was found in the cytoplasm [9]. These observations might explain why AEG-1 of predicted 64 kD molecule weight exhibits bands between 70-80 kD when detected by antibodies raised against various AEG-1 immunogens fragments. Nevertheless, thus far the significance of post-translational modification on the molecular function of AEG-1 remains to be understood.

## Elevated expression of AEG-1 in human cancers and other conditions

Elevation of AEG-1 expression has been appreciated in a spectrum of cancer types. Breast cancer, glioma and prostate cancer were among the first tumor types in which AEG-1 upregulation was recognized [6,7,10]. In breast cancer, it has found that the degree of AEG-1 expression correlates with clinical staging and tumor-node-metastasis (TNM) classification of the disease and might be useful as a potential prognostic marker [7]. Through copy number variation/gene expression association analysis, AEG-1 has been identified as a metastasis-associated gene whose overexpression is attributable to genomic amplification in breast cancer [11]. In esophageal squamous cell carcinoma, non-small cell lung cancer and hepatocellular carcinoma, the level of AEG-1 upregulation also correlates with clinical progression (staging) and poor prognosis of patients [12-14], further suggesting that AEG-1 might function as an oncogenic protein in various tumor types. Other tumor types in which upregulation of AEG-1 has been demonstrated include gastric cancer [15], colorectal carcinoma [16], osteosarcoma [17].

It is of note that in other conditions than cancer, altered expression of AEG-1 has also been reported. For example, an allele of rs1835740 near the AEG-1 locus is associated with migraine and increased AEG-1 expression [18]. Moreover, a recent study on the expression pattern of AEG-1 has indicated a possible role of AEG-1 in the development of brain, liver and skin [19]. In addition, AEG-1 could be induced by LPS stimulation of the U937 human promonocytic cells, suggesting a role of the molecule in toll-like receptor-mediated signaling [20].

AEG-1 overexpression in cancer has been attributed to an increase in the amount of its transcript resulting from one or both of two the following molecular mechanisms. Firstly, high AEG-1 expression in breast cancer and hepatocellular carcinoma is reportedly associated with genomic amplification of the AEG-1 locus [11,14]. The second mechanism, as suggested by several studies on the transcriptional regulation of the gene, is postulated based on the finding that AEG-1 mRNA is inducible by several regulatory signals. Activation of the Ras oncogene and subsequent induction of oncogenic transcription factor c-MYC leads to recruitment of c-MYC to the AEG-1 promoter region, which consequently transactivates AEG-1 expression [21]. Furthermore, transcription suppressor PLZF has been found to interact with AEG-1 and to facilitate the export of PLZF from the nucleus to cytoplasm, thereby freeing a binding region in the AEG-1 promoter/enhancer from the occupancy by the suppressive PLZF, leading to increased binding of transactivators, such as c-MYC, to its own promoter [8]. This postulated model provides a rational basis for the insight that a positive feedback loop might exist to support AEG-1 overexpression in pathological conditions. Pro-inflammatory factors including TNF-alpha and LPS could induce AEG-1 in both tumor and non-tumor cells [1,20]. While these stimuli are likely to activate inflammation-stimulated transcription factors such as NF kappa B and AP-1, the significance of AEG-1 in mediating the reciprocal interaction between inflammation and tumor development/progression remains to be determined. For most tumor types, the proportion of tumor samples with AEG-1 overexpression is significantly higher than that of those harboring AEG-1 amplification. Hence, it would be of great interest to further investigate whether cells harboring AEG-1 amplification seed a microenvironment that promotes AEG-1 expression throughout tumor progression.

## Biological functions of AEG-1

Interest in the effect of AEG-1 on the functions and the malignant phenotype of cancer cells is emerging as a hotspot in the field of cancer biology. Initial evidence demonstrated that overexpressing AEG-1 subjected breast cancer cells to lung homing [2], which was then further confirmed by *in vivo *experiments [11]. Furthermore, AEG-1 was demonstrated to promote invasion and migration of cancer cells. Emdad et al. first reported that the NF kappa B pathway was required for AEG-1-induced promotion of invasion of Hela cells *in vitro *[5]. In parallel, we found that AEG-1 promoted the invasion of glioma cells in mouse with an *in situ *xenotranplantion glioma model [22] through directly targeting and transactivating the promoter of MMP-9 gene, a molecule broadly recognized to be a major metalloproteinase required for ECM degradation and cytokine activation of invading cells during the processes of tumor invasion and metastasis, as well as other physiological and pathological conditions [23].

Another oncologically important notion is the possible involvement of AEG-1 in establishing chemoresistance, a hallmark of tumor aggressiveness [24]. In breast cancer, elevated AEG-1 enables tumor cells to escape cell death induced by paclitaxel, doxorubicin or cisplatin [11]. In this model, AEG-1-induced chemoresistance was thought to be mediated by survival-promoting genes, most notably ALDH3A1 and c-MET. In the same study, moreover, hydrogen peroxide-induced oxidative stress could also be relieved by AEG-1 downstream gene HOMOX1 [11]. Another investigation aimed to identify regulators of 5-FU resistance gene showed that AEG-1 promoted the expression of DPYD, the rate-limiting enzyme of 5-FU degradation [25]. AEG-1 has been shown to mediate upregulation of MDR1 via the PI3K/AKT signaling and augmentation of translation [26], a mechanism key to AEG-1-mediated efflux of doxorubicin. It is also noteworthy that AEG-1 overexpression is also associated with development of resistance in cells against other types of stress, such as hypoxia and glucose deprivation [27]. Moreover, evidence has shown that AEG-1 could trigger protective autophagy, which is a common mechanism employed by cancer cells to cope with metabolic stress. Recent studies have shown that by activating AMPK AEG-1 is able to elevate autophagy regulator ATG5 and lead to increased autophagy [28].

AEG-1 also contributes to tumor growth through pro-proliferative and anti-apoptotic effects. In prostate cancer [10] and breast cancer [29] elevation of AEG-1 has been clearly linked to downregulation of cell cycle inhibitors, leading to accelerated cell proliferation. Furthermore, in other models, AEG-1 has been found to be sufficient to suppress apoptosis through a systematic mechanism that upregulates apoptosis inhibitors via activation of the PI3K/Akt signaling pathway [30].

In many tumor types that display elevated AEG-1 expression, AEG-1 has been shown to enhance tumor-induced angiogenesis. In tumors formed by AEG-1-transduced rat embryo fibroblasts, intensive angiogenic markers including Ang-1, HIF1 alpha and CD31 are correlated with AEG-1 upregulation. It appears that the PI3K/AKT signaling is involved in AEG-1 induced angiogenesis [31]. Interestingly, in addition to its role in promoting the pro-angiogenic activity of tumor cells, endothelial AEG-1 might also be able to enhance the capability of vascular endothelial cells to form neovascularture, as ectopic expression of AEG-1 in HUVEC cells was found to result in increased tube formation [31]. Moreover, analysis of clinical samples taken from large cohorts of patients of breast cancer showed that AEG-1 correlated positively with the VEGF level and the microvascular density [32].

## Activation of oncogenic signal cascades by AEG-1

The molecular mechanisms underlying the oncogenic role of AEG-1 in human cancers has been studied during this decade. There has been a large abundance of evidence demonstrating that several major cellular signaling cascades might be associated with the ability of AEG-1 to execute the identified biological functions in various contexts. The NF kappa B pathway was first reported to be activated by AEG-1 through physical interaction with the p65 subunit of NF kappa B complex [5], with further evidence revealing that binding of AEG-1 to p65 resulted in recruitment of acetyltransferase CBP to p65, which might contribute to transcriptional activation [33]. It is believed that depending on the residue sets that are acetylated, the affinity with which p65 binds to I kappa B alpha can be increased or decreased, and thus the transcription activity of p65 is activated or inhibited correspondingly [34]. In the scenario of p65 activation induced by AEG-1, whether the recruitment of CBP results in acetylation of transactivating residues of p65 or alternatively other component(s) of the p65-containing complex that occupies the target promoter remains to be elucidated.

As aforementioned, in the attempt to understand the molecular mechanism that mediates the expression of AEG-1, the PI3K/AKT was identified to be required for Ras-induced AEG-1 expression [21]. Interestingly, the same study found that AKT was also a downstream mediator of the functions of AEG-1, as overexpression or silencing of AEG-1 resulted in elevation or attenuation, respectively, of the phosphorylation status of AKT [10,29,30]. Activation of AKT by AEG-1 led to phosphorylation of GSK3-beta and phosphorylation suppression of FOXO1/3 activity, resulting in enhanced cell survival and proliferation [10,29,30]. Given that PI3K/AKT signaling represents a commonly activated oncogenic pathway in various cancers and crosstalks with multiple signaling pathways, including NF kappa B [35], it is likely that AKT activation could further enhance the activation NF kappa B induced by AEG-1. In hepatocellular carcinoma, AEG-1 has been shown to increase phosphorylation of MAPK molecules, including ERK1/2 and p38, which subsequently activates Wnt-mediated signaling and consequently leads to increased tumor angiogenesis [14].

Interaction of AEG-1 with other proteins and the subsequent signaling effects remain to be understood. While several signaling pathways involved in mediating the oncogenic functions of AEG-1 have been elucidated, little is known about what direct interactions occur between AEG-1 and other proteins and how they contribute to the downstream effects of AEG-1.

## Engagement of AEG-1 in the RNA-induced silencing complex (RISC)

Recent reports by two independent groups have shown that AEG-1 could interact with components of the RISC complex. By employing yeast two-hybrid method, co-immunoprecipitation and mass-spectrometry, SND1 of the RISC complex was identified as a novel binding partner of AEG-1 [36,37]. SND1 was initially dentified as a transcription co-activator interacting with viral transcription factor EBNA2 [38] and as an activator of c-Myb [39] and STAT6 [40]. It was later reported to be contained in the RISC complex [41] and thus important for the editing and subsequent degradation of dsRNA [42]. Disruption of SND1 in *C. elegance *results in defects in the action of siRNA [41], which underscores its importance in RNAi-induced gene silencing. As AEG-1 has been demonstrated to interact with SND1 and enhance the activity of RISC [36], it increases degradation of tumor suppressor mRNAs that are target of onco-miRs [37]. What remains interesting is that AEG-1 mediated-activation of RISCs appears to be a general observation for all RISCs in cells with high AEG-1 expression, and yet it is unclear whether the net oncogenic effects of microRNAs documented in cancer cells are due to a selective interaction between AEG-1 and onco-miR-containing RISCs. Alternatively, AEG-1 may facilitate the function of all RISCs in a non-selective manner, and the oncogenic effects of microRNAs merely result from the fact that tumor cells harbor onco-miRs at higher levels than those of tumor suppressive microRNAs. Nevertheless, despite that the significance of the interactions between AEG-1 and RISC demands further investigation, the finding of AEG-1 as a component of RISC might provide new insights in how AEG-1 promotes a wide variety of oncogenic activities at a highly context-dependent fashion. To understand how AEG-1 affects RISC activity, studies on whether AEG-1 could regulate RNA recognition or cleavage activity are essential.

## Summary and perspectives

**Figure 1:**
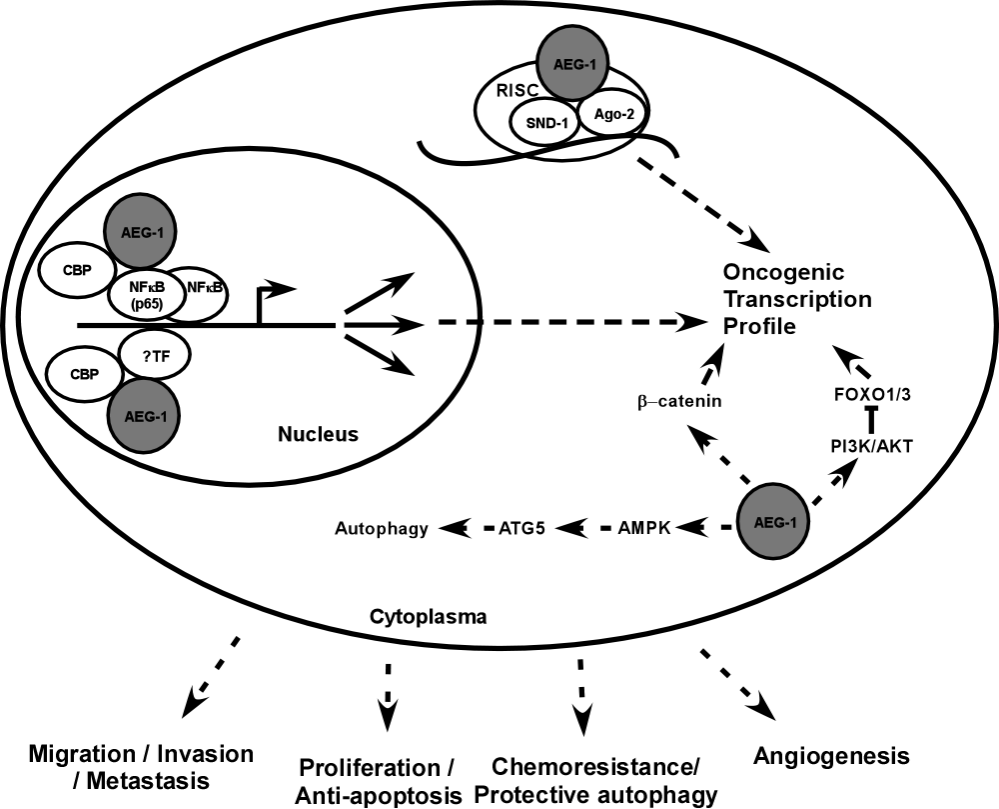
**A model illustrating the biological functions and possible underlying molecular mechanisms of AEG-1**. Dashed arrows indicate indirect molecular effects.

Accumulating evidence has shown that AEG-1 plays remarkable roles in the regulation of various physiological and pathological processes [43]. A model presented as Figure 1 attempts to illustrate possible molecules and pathways that have been suggested by mechanistic studies to mediate the biological functions of this important protein. Although a great abundance of literature has documented an array of cellular functions associated with altered expression level of AEG-1, we are yet far from revealing the biological significance of this molecule in physiological and pathological conditions. Experimental evidence from AEG-1 knockout or transgenic animal models would be important for further understanding of the functional significance of AEG-1 in embryonic development and diseases. Evolutionally, AEG-1 is only found in vertebrates that have complete immune systems developed, and it is noteworthy that the signaling pathways identified thus far modulated by AEG-1 have also been associated with the regulation of inflammation and immune response. It is thus of importance to further investigate the significance of aberrated AEG-1 expression in pathogenesis related to abnormal immune response or nonresolving inflammation, such as cancer, autoimmune diseases and severe immunopathological conditions caused by pathogen infection. At the molecule level, evidence is still needed to illustrate whether AEG-1 exerts its biological function by biochemically modifying its binding partners or alternatively, by acting as a scaffold of the signaling/effector complex. Moreover, the biochemical modification occurring to AEG-1 itself is also of great interest for further investigation, as such modification(s), if proved to be functionally important for AEG-1, would be potentially promising targets for optimal inhibitors or therapeutic drugs against diseases involving upregulated or over-activated AEG-1, including cancer.

AEG-1 is also under investigation and development for its potential value as a diagnostic or prognostic biomarker based on its overexpression and correlations with disease staging and outcome throughout a wide range of cancer types. Yet for the latter application, however, larger cohorts of patient studies and prospective investigation on the correlation of AEG-1 mRNA or protein in blood, urine and biopsy samples with clinical characteristics are urgently needed.

## Competing interests

The authors declare that they have no competing interests.

## Authors' contributions

ZY, JL and MFL wrote the review. All authors read and approved the final manuscript.

## References

[B1] Su ZZ Kang DC Chen Y Pekarskaya O Chao W Volsky DJ Fisher PB Identification and cloning of human astrocyte genes displaying elevated expression after infection with HIV-1 or exposure to HIV-1 envelope glycoprotein by rapid subtraction hybridization, RaSH Oncogene 2002 21 3592 3602 10.1038/sj.onc.1205445 12032861

[B2] Brown DM Ruoslahti E Metadherin, a cell surface protein in breast tumors that mediates lung metastasis Cancer Cell 2004 5 365 374 10.1016/S1535-6108(04)00079-0 15093543

[B3] Britt DE Yang DF Yang DQ Flanagan D Callanan H Lim YP Lin SH Hixson DC Identification of a novel protein, LYRIC, localized to tight junctions of polarized epithelial cells Exp Cell Res 2004 300 134 148 10.1016/j.yexcr.2004.06.026 15383321

[B4] Sutherland HG Lam YW Briers S Lamond AI Bickmore WA 3D3/lyric: a novel transmembrane protein of the endoplasmic reticulum and nuclear envelope, which is also present in the nucleolus Exp Cell Res 2004 294 94 105 10.1016/j.yexcr.2003.11.020 14980505

[B5] Emdad L Sarkar D Su ZZ Randolph A Boukerche H Valerie K Fisher PB Activation of the nuclear factor kappaB pathway by astrocyte elevated gene-1: implications for tumor progression and metastasis Cancer Res 2006 66 1509 1516 10.1158/0008-5472.CAN-05-3029 16452207

[B6] Emdad L Sarkar D Su ZZ Lee SG Kang DC Bruce JN Volsky DJ Fisher PB Astrocyte elevated gene-1: recent insights into a novel gene involved in tumor progression, metastasis and neurodegeneration Pharmacol Ther 2007 114 155 170 10.1016/j.pharmthera.2007.01.010 17397930 PMC2039930

[B7] Li J Zhang N Song LB Liao WT Jiang LL Gong LY Wu J Yuan J Zhang HZ Zeng MS Li M Astrocyte elevated gene-1 is a novel prognostic marker for breast cancer progression and overall patient survival Clin Cancer Res 2008 14 3319 3326 10.1158/1078-0432.CCR-07-4054 18519759

[B8] Thirkettle HJ Mills IG Whitaker HC Neal DE Nuclear LYRIC/AEG-1 interacts with PLZF and relieves PLZF-mediated repression Oncogene 2009 28 3663 3670 10.1038/onc.2009.223 19648967

[B9] Thirkettle HJ Girling J Warren AY Mills IG Sahadevan K Leung H Hamdy F Whitaker HC Neal DE LYRIC/AEG-1 is targeted to different subcellular compartments by ubiquitinylation and intrinsic nuclear localization signals Clin Cancer Res 2009 15 3003 3013 10.1158/1078-0432.CCR-08-2046 19383828

[B10] Kikuno N Shiina H Urakami S Kawamoto K Hirata H Tanaka Y Place RF Pookot D Majid S Igawa M Dahiya R Knockdown of astrocyte-elevated gene-1 inhibits prostate cancer progression through upregulation of FOXO3a activity Oncogene 2007 26 7647 7655 10.1038/sj.onc.1210572 17563745

[B11] Hu G Chong RA Yang Q Wei Y Blanco MA Li F Reiss M Au JL Haffty BG Kang Y MTDH activation by 8q22 genomic gain promotes chemoresistance and metastasis of poor-prognosis breast cancer Cancer Cell 2009 15 9 20 10.1016/j.ccr.2008.11.013 19111877 PMC2676231

[B12] Yu C Chen K Zheng H Guo X Jia W Li M Zeng M Li J Song L Overexpression of astrocyte elevated gene-1 (AEG-1) is associated with esophageal squamous cell carcinoma (ESCC) progression and pathogenesis Carcinogenesis 2009 30 894 901 10.1093/carcin/bgp064 19304953

[B13] Song L Li W Zhang H Liao W Dai T Yu C Ding X Zhang L Li J Over-expression of AEG-1 significantly associates with tumour aggressiveness and poor prognosis in human non-small cell lung cancer J Pathol 2009 219 317 326 10.1002/path.2595 19644957

[B14] Yoo BK Emdad L Su ZZ Villanueva A Chiang DY Mukhopadhyay ND Mills AS Waxman S Fisher RA Llovet JM Astrocyte elevated gene-1 regulates hepatocellular carcinoma development and progression J Clin Invest 2009 119 465 477 10.1172/JCI36460 19221438 PMC2648696

[B15] Jian-Bo X Hui W Yu-Long H Chang-Hua Z Long-Juan Z Shi-Rong C Wen-Hua Z Astrocyte-elevated gene-1 overexpression is associated with poor prognosis in gastric cancer Med Oncol 2011 28 455 462 10.1007/s12032-010-9475-6 20300973

[B16] Song H Li C Li R Geng J Prognostic significance of AEG-1 expression in colorectal carcinoma Int J Colorectal Dis 2010 25 1201 1209 10.1007/s00384-010-1009-3 20625905

[B17] Wang F Ke ZF Sun SJ Chen WF Yang SC Li SH Mao XP Wang LT Oncogenic roles of astrocyte elevated gene-1 (AEG-1) in osteosarcoma progression and prognosis Cancer Biol Ther 2011 12 10.4161/cbt.12.6.16301 21750404

[B18] Anttila V Stefansson H Kallela M Todt U Terwindt GM Calafato MS Nyholt DR Dimas AS Freilinger T Muller-Myhsok B Genome-wide association study of migraine implicates a common susceptibility variant on 8q22.1 Nat Genet 2010 42 869 873 10.1038/ng.652 20802479 PMC2948563

[B19] Jeon HY Choi M Howlett EL Vozhilla N Yoo BK Lloyd JA Sarkar D Lee SG Fisher PB Expression patterns of astrocyte elevated gene-1 (AEG-1) during development of the mouse embryo Gene Expr Patterns 2010 10 361 367 10.1016/j.gep.2010.08.004 20736086 PMC3165053

[B20] Khuda II Koide N Noman AS Dagvadorj J Tumurkhuu G Naiki Y Komatsu T Yoshida T Yokochi T Astrocyte elevated gene-1 (AEG-1) is induced by lipopolysaccharide as toll-like receptor 4 (TLR4) ligand and regulates TLR4 signalling Immunology 2009 128 e700 706 10.1111/j.1365-2567.2009.03063.x 19740331 PMC2753960

[B21] Lee SG Su ZZ Emdad L Sarkar D Fisher PB Astrocyte elevated gene-1 (AEG-1) is a target gene of oncogenic Ha-ras requiring phosphatidylinositol 3-kinase and c-Myc Proc Natl Acad Sci USA 2006 103 17390 17395 10.1073/pnas.0608386103 17088530 PMC1859939

[B22] Liu L Wu J Ying Z Chen B Han A Liang Y Song L Yuan J Li J Li M Astrocyte elevated gene-1 upregulates matrix metalloproteinase-9 and induces human glioma invasion Cancer Res 2010 70 3750 3759 10.1158/0008-5472.CAN-09-3838 20388776

[B23] Kessenbrock K Plaks V Werb Z Matrix metalloproteinases: regulators of the tumor microenvironment Cell 2010 141 52 67 10.1016/j.cell.2010.03.015 20371345 PMC2862057

[B24] Hanahan D Weinberg RA Hallmarks of cancer: the next generation Cell 2011 144 646 674 10.1016/j.cell.2011.02.013 21376230

[B25] Yoo BK Gredler R Vozhilla N Su ZZ Chen D Forcier T Shah K Saxena U Hansen U Fisher PB Sarkar D Identification of genes conferring resistance to 5-fluorouracil Proc Natl Acad Sci USA 2009 106 12938 12943 10.1073/pnas.0901451106 19622726 PMC2722317

[B26] Yoo BK Chen D Su ZZ Gredler R Yoo J Shah K Fisher PB Sarkar D Molecular mechanism of chemoresistance by astrocyte elevated gene-1 Cancer Res 2010 70 3249 3258 10.1158/0008-5472.CAN-09-4009 20388796 PMC2855753

[B27] Noch E Bookland M Khalili K Astrocyte-elevated gene-1 (AEG-1) induction by hypoxia and glucose deprivation in glioblastoma Cancer Biol Ther 2011 11 32 39 10.4161/cbt.11.1.13835 21084864 PMC3047099

[B28] Bhutia SK Kegelman TP Das SK Azab B Su ZZ Lee SG Sarkar D Fisher PB Astrocyte elevated gene-1 induces protective autophagy Proc Natl Acad Sci USA 2010 107 22243 22248 10.1073/pnas.1009479107 21127263 PMC3009793

[B29] Li J Yang L Song L Xiong H Wang L Yan X Yuan J Wu J Li M Astrocyte elevated gene-1 is a proliferation promoter in breast cancer via suppressing transcriptional factor FOXO1 Oncogene 2009 28 3188 3196 10.1038/onc.2009.171 19633686

[B30] Lee SG Su ZZ Emdad L Sarkar D Franke TF Fisher PB Astrocyte elevated gene-1 activates cell survival pathways through PI3K-Akt signaling Oncogene 2008 27 1114 1121 10.1038/sj.onc.1210713 17704808

[B31] Emdad L Lee SG Su ZZ Jeon HY Boukerche H Sarkar D Fisher PB Astrocyte elevated gene-1 (AEG-1) functions as an oncogene and regulates angiogenesis Proc Natl Acad Sci USA 2009 106 21300 21305 10.1073/pnas.0910936106 19940250 PMC2795510

[B32] Li C Li R Song H Wang D Feng T Yu X Zhao Y Liu J Wang Y Geng J Significance of aeg-1 expression in correlation with vegf, microvessel density and clinicopathological characteristics in triple-negative breast cancer J Surg Oncol 2010 10.1002/jso.21788 21259255

[B33] Sarkar D Park ES Emdad L Lee SG Su ZZ Fisher PB Molecular basis of nuclear factor-kappaB activation by astrocyte elevated gene-1 Cancer Res 2008 68 1478 1484 10.1158/0008-5472.CAN-07-6164 18316612

[B34] Glozak MA Sengupta N Zhang X Seto E Acetylation and deacetylation of non-histone proteins Gene 2005 363 15 23 16289629 10.1016/j.gene.2005.09.010

[B35] Vivanco I Sawyers CL The phosphatidylinositol 3-Kinase AKT pathway in human cancer Nat Rev Cancer 2002 2 489 501 10.1038/nrc839 12094235

[B36] Yoo BK Santhekadur PK Gredler R Chen D Emdad L Bhutia S Pannell L Fisher PB Sarkar D Increased RNA-induced silencing complex (RISC) activity contributes to hepatocellular carcinoma Hepatology 2011 53 1538 1548 10.1002/hep.24216 21520169 PMC3081619

[B37] Blanco MA Aleckovic M Hua Y Li T Wei Y Xu Z Cristea IM Kang Y Identification of Staphylococcal Nuclease Domain-containing 1 (SND1) as a Metadherin-interacting Protein with Metastasis-promoting Functions J Biol Chem 2011 286 19982 19992 10.1074/jbc.M111.240077 21478147 PMC3103372

[B38] Tong X Drapkin R Yalamanchili R Mosialos G Kieff E The Epstein-Barr virus nuclear protein 2 acidic domain forms a complex with a novel cellular coactivator that can interact with TFIIE Mol Cell Biol 1995 15 4735 4744 7651391 10.1128/mcb.15.9.4735 PMC230717

[B39] Leverson JD Koskinen PJ Orrico FC Rainio EM Jalkanen KJ Dash AB Eisenman RN Ness SA Pim-1 kinase and p100 cooperate to enhance c-Myb activity Mol Cell 1998 2 417 425 10.1016/S1097-2765(00)80141-0 9809063

[B40] Valineva T Yang J Palovuori R Silvennoinen O The transcriptional co-activator protein p100 recruits histone acetyltransferase activity to STAT6 and mediates interaction between the CREB-binding protein and STAT6 J Biol Chem 2005 280 14989 14996 10.1074/jbc.M410465200 15695802

[B41] Caudy AA Ketting RF Hammond SM Denli AM Bathoorn AM Tops BB Silva JM Myers MM Hannon GJ Plasterk RH A micrococcal nuclease homologue in RNAi effector complexes Nature 2003 425 411 414 10.1038/nature01956 14508492

[B42] Scadden AD The RISC subunit Tudor-SN binds to hyper-edited double-stranded RNA and promotes its cleavage Nat Struct Mol Biol 2005 12 489 496 10.1038/nsmb936 15895094

[B43] Yoo BK Emdad L Lee SG Su ZZ Santhekadur P Chen D Gredler R Fisher PB Sarkar D Astrocyte elevated gene-1 (AEG-1): A multifunctional regulator of normal and abnormal physiology Pharmacol Ther 2011 130 1 8 10.1016/j.pharmthera.2011.01.008 21256156 PMC3043119

